# Investigating male gamers' behavioral intention to play PUBG: Insights from playful-consumption experiences

**DOI:** 10.3389/fpsyg.2022.909875

**Published:** 2022-08-18

**Authors:** Umair Rehman, Muhammad Umair Shah, Amir Zaib Abbasi, Helmut Hlavacs, Rameen Iftikhar

**Affiliations:** ^1^User Experience Design, Wilfrid Laurier University, Brantford, ON, Canada; ^2^University of Waterloo, Waterloo, ON, Canada; ^3^IRC for Finance and Digital Economy, KFUPM Business School, King Fahd University of Petroleum and Minerals, Dhahran, Saudi Arabia; ^4^University of Vienna, Vienna, Austria; ^5^Lahore University of Management Sciences, Lahore, Pakistan

**Keywords:** Battle Royal games, hedonic consumption experience, playful-consumption experiences, uses and gratifications theory, gaming, entertainment computing

## Abstract

This research investigates the factors that affect male gamers' behavioral intention to play PlayerUnknown's Battlegrounds (PUBG), which is one of the most widely played online games of today's era. We examine the factors through the lens of the hedonic consumption model (i.e., playful-consumption experiences) and use the gratification theory to predict behavioral intention to play PUBG. Data from 248 male PUBG gamers were analyzed using PLS-SEM analyses. The study involved an initial stage where an estimation model (i.e., measurement model) was analyzed to assess the constructs' reliability and validity. Following this, the second stage involved assessing the theoretical model to test the relationship between the principle constructs. The study found that playful-consumption experience factors, such as escapism, emotional involvement, sensory experience, enjoyment, and arousal, significantly influenced the behavioral intentions to play PUBG. The research findings further indicate that role-projection and fantasy failed to impact consumers' intention to play PUBG. This study provides both theoretical and practical implications. It fills the literature gap by focusing on predicting the behavioral intention to play PUBG through the playful-consumption experiences of a popular online multiplayer game. Practically, this study could potentially open avenues for gaming companies to address how different playful-consumption experiences impact game users' behavioral intentions.

## Introduction

Information system (I.S) research has initially concentrated on productivity-oriented I.S. (Wu and Holsapple, 2014), which consists of technology that is intended to reach productive outcomes such as word-processing, simulations, and computations (Adams et al., 1992; Karahanna et al., 1999; Venkatesh and Davis, 2000; Wang, 2003). However, there has been an increasing interest in research toward technology intended for entertainment and leisure purposes known as pleasure-oriented I.S. With the rise of pleasure-oriented I.S., the popularity of playing online, multiplayer videogames using mobile phones, computers (P.C.), consoles (e.g., PS4) (Abbasi et al., 2019a), and other portable mediums has also increased significantly in the last decade (Lee, 2009). The widespread availability of videogames on different platforms has allowed consumers from diverse socioeconomic and cultural demographics to experience enduring entertainment value (Allaire et al., 2013).

Within games, online multiplayer games have been garnering particular interest due to their widespread use (Sourmelis et al., 2017; Wang et al., 2020). For instance, the Multiplayer Online Battle Arena (MOBA) games, such as League of Legends, Dota 2, and Arena of Valor, among others, have been unusually popular (Sourmelis et al., 2017; Mora-Cantallops and Sicilia, 2018; Abbasi et al., 2020a; Kordyaka et al., 2020). There exists an opportunity to advance research in this area by researching a single game to uncover the hedonistic attributes that lead to the formulation of behavioral intention for its use. We select the PlayerUnknown's Battlegrounds (PUBG) game to investigate this premise. PUBG is a Battle Royale (BR) game that individuals of ages 16 years and above can play on different mediums, such as computers (P.C.), consoles, or mobiles. BR genre is best described by gameplay features, such as survival and exploration; within BR games, players are generally driven by this concept of “last-man standing,” which encourages them to be the ultimate contender in the game (Fernandez de Henestrosa et al., 2022). This gameplay feature also leads to increased videogame engagement and long-term player retention (Fernandez de Henestrosa et al., 2022). PUBG is one of the most popular games of the modern era that witnessed over 3.24 million concurrent players in January 2018 and reached 50 million in unit sales by January 2018 (Gough, 2020). As a result, PUBG has amassed many accolades, including the Best Multiplayer Game at The Game Awards in 2017 (Gough, 2020).

Given this rise in popularity, the formulation of behavioral intention has been studied quite intensively in Information Systems (I.S.) research, where researchers have focused on addressing questions related to why people use specific technology products (Davis, 1989). Behavioral intention is grounded in the Theory of Planned Behavior (TPB), and the theory posits that the stronger an intention to perform a behavior, such as using a certain form of I.S., the higher will be the likelihood that the user will truly perform that behavior (Jackson et al., 1997). Investigating the behavioral intention of users allows researchers to uncover the motivational factors that lead to the actual use of a product or a service (Netemeyer and Bearden, 1992). Such an investigation carries far-reaching implications for companies designing products for positive user experience, which lead to long-term usage and customer retention.

In videogame settings, many researchers investigated the underlying factors of videogame play (Selnow, 1984; Wigand et al., 1986; Phillips et al., 1995; Vorderer et al., 2003; Sherry et al., 2012). Specifically, attention has been spread to the relationship between videogame play and behavioral attention (Sherry et al., 2012). The Theory of Reasoned Action (TRA) helps gauge an individual's attitude toward use, their behavioral intention toward use, and their actual practical use of innovation (Davis, 1989), by exploring their perceived usefulness and ease of use of said innovation (Fishbein and Ajzen, 1977). From the TRA, the Technology Acceptance Model (TAM) can be derived which further establishes a relationship between perceived usefulness and behavioral intention which hinges upon the user's acceptance and reliance on technology (Davis, 1989). These two models have been used to associate behavioral intention with increased use of technology and acceptance of innovation (Lee et al., 2009). However, research into the behavioral intentions behind playing multi-player games has been limited while these games have gained immense popularity. In specific, PUBG continues to be one of the most popular games available on Steam (Hollebeek et al., 2022).

According to the uses and gratifications theory (UGT), we can uncover factors that explain why people seek certain media to satisfy their needs (Ruggiero, 2000). UGT is also grounded in the hedonic consumption framework that informs us that consumers use media sources, such as videogames, social media, and television, to satisfy user needs, comprising social and personal integrative, affective, tension-release needs, and cognitive (Settle, 2018). Even though UGT was originally applied to radio broadcasts, television transmissions, and newspapers, its application has been extended to different contexts, including social networking sites (Rathnayake and Winter, 2018), location-based games (Hamari et al., 2019), eSports (Hamari and Sjöblom, 2017), social network games (Huang et al., 2017), and online games (Li et al., 2015). Researchers have become increasingly convinced by UGT's application; thus, they have now started to apply it as a foundational framework to understand players' behavioral intention and usage patterns by exploring playful-consumption experience factors (Abbasi et al., 2021a). Irrespective of the medium to which UGT is applied, old media or new media, the reasons behind their continuous use remain similar (Chang and Lee, 2022). This could partly be because of the five assumptions upon which UGT rests, namely audience of the media source is active and goal-oriented, the audience member chooses the media source to gratify themselves, the media source is competitive with other sources for need satisfaction, audience is aware of their media usage, and the audiences' ability to provide an accurate data about their use and value judgement of their media source (Ma, 2021). However, the existing research has only investigated the three hedonic factors from UGT, namely escapism, enjoyment, and fantasy, and has ignored playful-consumption experience factors, such as sensory experience, role-projection, emotional involvement, and arousal.

The hedonic consumption theory posits that users engage with technology to satisfy their needs (Hirschman and Holbrook, 1982). With pleasure-oriented I.S., the user's need is primarily entertainment. The use of this technology meets the needs for the user's gratification through its hedonic characteristics. This influences continued usage, as explained under the UGT, due to its provision of pleasurable service. Hedonic consumption differs significantly from the utilitarian view. The utilitarian view promotes a perspective that relates to the use of products or services for practical or constructive purposes. People use these productivity-oriented I.S. mainly due to their perceived usefulness (P.U.) (Wu and Du, 2012) and perceived ease of use (PEOU) (Van der Heijden, 2004); however, with the focus on videogames, hedonic aspects of technology need to be taken into account (Chang et al., 2014).

One of the aims of the current research is to explore how determinants of behavioral intentions for entertainment-related I.S. can be mapped through specific hedonic and playful-consumption experience factors. As discussed previously, TAM variables, such as P.U. and PEOU, are less relevant for hedonic forms of I.S., whereas predictor variables for TRA only offer a generic characterization at a foundational level. We aim to capture specific aspects within the PUBG, which impact players' behavioral intentions and lead to sustained usage. While TRA predictors are valuable when capturing general insights on attitudes and norms within a given context, given the specialized focus and the context of the study, TRA predictors are too broad to effectively capture specific facets of player experience which impact behavioral intention.

While there has been adequate research that aims to analyze the processes of play on a generic level, research exploring the role of experiential aspects on play processes is limited. In-game experiences are central to understanding the creative processes gamers employ to succeed in simulated environments. The experiential processes of play further uncover dynamics, which lead to sustained engagement. In the current research, we specifically cater to imaginal, emotional, and sensory experiences of the PUBG game, which are crucial to understanding the behavioral patterns of players and their interaction in virtual and synthetic environments.

In the light of the hedonic consumption theory and UGT, there is a need to investigate how playful-consumption experiences, in addition to the previously investigated hedonic factors, affect consumers' behavioral intentions to use products and services that are generally pleasure-oriented, such as videogames. The hedonic consumption theory has been explored in terms of the multisensory, fantasy, and emotive traits associated with the use of technology for gratification (Abbasi et al., 2020c; Hollebeek et al., 2022). We have distilled factors from these categories to create a model that is related to the player's experiences, particularly their intention to keep engaging with the pleasure-oriented I.S. In this research, we explore hedonic and playful-consumption experience factors to uncover the experiences that lead to the formulation of users' behavioral intention to play PUBG.

In the light of the hedonic consumption framework and other relevant findings, we posit that experiences, such as imaginal, emotional, and sensory experiences, are crucial in formulating behavioral intention to play PUBG. We suggest that imaginal experiences are shaped through experiences, such as escapism, fantasy, and role-projection. Additionally, emotional experiences are affected by factors, such as enjoyment, emotional involvement, and arousal. Moreover, the visual stimuli within PUBG affect a player's sensory experience. Only through a detailed investigation, one can ascertain which of the hedonic consumption and playful experience factors tangibly lead to the formulation of behavioral intention to play PUBG.

## Research model and hypotheses development

Building upon the hedonic consumption perspective and other relevant theories, we believe that imaginal, emotional, and sensory experiences influence users' behavioral intention to play the PUBG game.

### Imaginal experience

Imaginal experience is defined as the psychological act of visualizing ideas that do not have an existential reality. For the scope of this study, we define imaginal experiences to include fantasy, role-projection, and escapism (Abbasi et al., 2019a).

**Escapism** is the tendency to escape from the uncomfortable reality and distract one's self from real-life issues (Henning and Vorderer, 2001). In this time and space, escapism is becoming more and more important for the day-to-day functioning of most people (Hirschman, 1983). People engage in pleasure-seeking behavior, ranging from music to movies and TV shows, to escape from their environment (Henry and Caldwell, 2006). Online gaming encourages users toward finding an escape (Hoon et al., 2002; Shin, 2019). Barton (2008) found out that to find relief from real-life problems, people pursue activities such as online gaming. PUBG offers a synthetic environment that can be a powerful source of escape for its users especially due to the immersive nature of the game. Therefore, this study hypothesizes that:

**H1:** Escapism has a positive influence on the player's intention to play PUBG games.

**Fantasy** is the process of creating an alternate reality that tries to satisfy an individual's emotional needs (Hollebeek et al., 2022). The process of forming a fantasy takes place when people undergo high levels of excitement. This is critical in BR games, as it provides an avenue for a two-way response of a person who increasingly fantasizes about game-playing. Fantasy aims to build a reality whose foundations lie in creating an interesting, engaging, and believable world, the construction of which may vary from person to person. This explains why individuals who want to escape their reality and plunge into an alternate world play PUBG games. Bormann (2001) points out that fantasy helps individuals in visualizing an ideal world. Instead of limiting and restricting possibilities and impossibilities, fantasy can create the desired “unreality” where the impossible can be achieved (Martin, 2004). This constructed “unreality” helps people live better lives (Henry and Caldwell, 2006). This can be achieved through indulging in games that appeal to their fantasies, made possible via immersive plots, virtual reality, and audio-visual aids. This is why players spend a lot of time playing online games since it helps them prolong their stay in their imaginary worlds (Dormans, 2006). We believe that this could be one of the reasons behind their involvement with PUBG games. Therefore, this study hypothesizes that:

**H2:** Fantasy has a positive influence on the player's intention to use PUBG games.

**Role projection** refers to the individual's ability to project themselves onto a character or a particular role (Hirschman, 1983; Wu and Holsapple, 2014). In particular, videogames allow gamers to project their selves in a specific role and act accordingly in virtual settings (Abbasi et al., 2021a). Online platforms for entertainment, particularly the PUBG, are an effective medium for role projection due to the context it offers to its audience. Besides, PUBG offers roles to gamers' that they adopt and make a team of five gamers to fight against each other and each member of the team is assigned a specific role to follow so that they can together win the game. Given that, the gaming environment allows players to mentally project themselves into different and specific roles to collectively work on winning the game (i.e., achieving the chicken dinner; Aggarwal et al., 2020). Hence, this study proposes that:

**H3:** Role projection has a positive influence on the player's intention to play PUBG games.

### Emotional experience

Emotional experiences are the feelings that an individual experiences as a response to certain stimuli (Lacher and Mizerski, 1994; Lee et al., 2009). The different emotional experiences include enjoyment, emotional involvement, and arousal (Hirschman and Holbrook, 1982; Stewart, 2013; Wu and Holsapple, 2014).

**Enjoyment** defines as an individual's experience of pleasure and joy by participating in an activity (Abbasi et al., 2019b). Enjoyment does not limit to physical actions, for example, dancing and exercising but can also be extended to mental activities such as playing games. Van der Heijden (2004) further explains this relationship between enjoyment and intended continued use in his study, where he concluded that enjoyment is an essential hedonic latent construct. Hence, it plays a significant role in an individual's motivation (or intention) to keep using the technology that provides entertainment, and online gaming is one of these. Existing literature also corroborates this by pointing out that enjoyment is an indicator that encourages gameplay (Davis et al., 2005). Various gameplay elements such as collaborating with other players, strategizing to win against the common opponent, and audio-visual feedback make this possible. In specific, this may be one of the reasons behind the player's intention to continue indulging in PUBG gameplay. Therefore, it is hypothesized that:

**H4:** Enjoyment has a positive influence on the player's intention to play PUBG games.

**Emotional involvement** is a psychologically charged situation when an individual is performing a specific behavior or activity (Abbasi et al., 2019a). The behavior may differ in terms of intensity and duration. Emotional involvement in videogames (e.g., PUBG) takes place when players are highly engaged in their videogame playing that has high intensity, long-lasting, and motivating (Hollebeek et al., 2022). Studies show that when people get emotionally invested in a situation, they often forget about their reality as a consequence (Lacher and Mizerski, 1994). This can also be seen in immersive game-playing (Lugmayr and Teras, 2015). As a result, high levels of emotional involvement are present in PUBG games, which stimulates players' intention to play the PUBG game. On this basis, we posit that:

**H5:** Emotional Involvement has a positive influence on the player's intention to play PUBG games.

**Arousal** refers to an emotional state similar to alertness, emotional activation, and excitement due to external factors (Hollebeek et al., 2022). Arousal is an individual's physiological and psychological state, which aids in activating emotions. Lacher and Mizerski (1994) evidenced that arousal is an important factor in predicting users' intention to purchase a product. Abbasi et al. (2021a) found that gamers' perceived arousal state positively explains the gamers' intention to continue playing the game (e.g., BR). Given that, if a player touches the experience of arousal state, it provides them an enjoyable and motivating experience to further involve in the gaming activity. This study assumes that the state of arousal experienced by PUBG players may persuade them to play frequently. Therefore, we state that:

**H6:** Arousal has a positive influence on the player's intention to play PUBG games.

### Sensory experience

Hirschman and Holbrook (1982) describe a sensory experience as “the receipt of involvement in various tangible modalities containing sound, touch, and sight.” The sensory attributes of online gaming may determine the gamer's future behavior and attitude toward gaming pertaining to audio esthetics, operational learning, graphics, and haptic dimensions as stated by Mason (2017) and the theory of flow put forward by Csikszentmihalyi and Csikszentmihalyi (1992). In terms of PUBG, these stimuli can be found through gameplay elements such as the soundtrack, sound effects, visual and graphic elements, and overall esthetic of the game. Therefore, we bring forward that:

**H7:** Sensory experience has a positive influence on the player's intention to play PUBG games.

## Research methodology

We used the cross-sectional survey approach as we focused on predicting the role of playful-consumption experiences of PUBG gamers on the outcome variable, that is, behavioral intention (Salkind, 2010). Besides, this approach is relatively fast, easy, and economical when it comes to conducting studies to verify new theories or proposed relationships (Sedgwick, 2014). Following a cross-sectional approach, we designed the study questionnaire into two main parts. The first part included demographic details of respondents such as gender, age, and education and their consumption patterns relating to PUBG games, for instance, frequency and money spent on gaming and accessories. Whereas, the second part covered the study variables and their associated items adapted from reliable and valid sources. The items measuring the playful-consumption experiences comprising emotional involvement, fantasy, escapism, role-projection, enjoyment, arousal, and sensory experience were adapted (Abbasi et al., 2017, 2019a; Hollebeek et al., 2022). The items assessing the construct of behavioral intention were adapted from Wu and Holsapple (2014), see Appendix A for the detailed particulars of the study's questionnaire.

To establish an appropriate sample size for the study, we employed the G^*^power analysis tool by Faul et al. (2007). It helped us calculate the minimum required sample based on our study model. Using the G^*^power tool, we gave the input parameters: the effect size is (f^2^) = 0.15, α error probability = 0.05, power (1-β err probability) = 0.95, and predictors = 7. As a result, we found that the minimum required sample was 153 to perform PLS-SEM analyses.

We employed a standard survey design method known as the online self-administered questionnaire. Purposive sampling was employed since it is a reliable sampling technique used in marketing and social sciences research (Etikan et al., 2016). Purposive sampling is essentially a non-probability sampling approach (Abbasi et al., 2021b), where the researcher applies individual judgment to choose the representative participant population, which is compatible with the aims and objectives of the study. In the current context, purposive sampling allows us to achieve greater access to our target audience for digital gaming.

We selected PUBG gamers that predominantly fall between the cohorts of generation Z and generation Y (Dimock, 2019). With regard to distribution, we issued ~450 survey invitations using a wide variety of social media platforms. For example, we used WhatsApp and Facebook groups to reach the intended audience of PUBG gamers.

We received 350 responses from our study respondents. Following preliminary checks, which included assessing the data for biases and incomplete responses, the final sample yielded 294 valid cases. While checking the data, we found that there were only 46 females out of 294 data, and hence considered only male gamers (i.e., 248) to generalize the study findings on male PUBG gamers only. Our data exceeded the recommended sample size (i.e., calculated through G^*^power). Table 1 provides a detailed overview of the respondents' profiles.

**Table 1:** Respondents' detail.

**Gender**	**%**
Male	100
**Age**	
16–20 years	21.0
21–25 years	33.9
26–30 years	31.9
31–35 years	7.3
36–40 years	6.0
**Education**	
Secondary school	10.9
Diploma/Higher secondary school	18.5
Undergraduate	36.3
Postgraduate	34.3
**Gaming device**	
Personal computer	25.0
Dedicated-gaming console	0.8
Smartphone	71.8
Other	2.4
**Gaming frequency**	
Everyday	57.3
Few times a week	32.7
Once a week	10.1
**Time spent on PUBG game**	
1 h	4.8
2 h	19.4
3 h	25.4
4 h and above	50.4

Data from a cross-sectional survey design related to a single source (here: sample subject) create concerns about common method bias. To determine whether the study data are affected by common method bias, we performed Harmon's single-factor analysis (Podsakoff et al., 2003). The research findings suggest that a single factor explains the 39% variance. Therefore, our results were not biased and met the quality threshold, maintaining a variance of <0.50.

**Figure 1:**
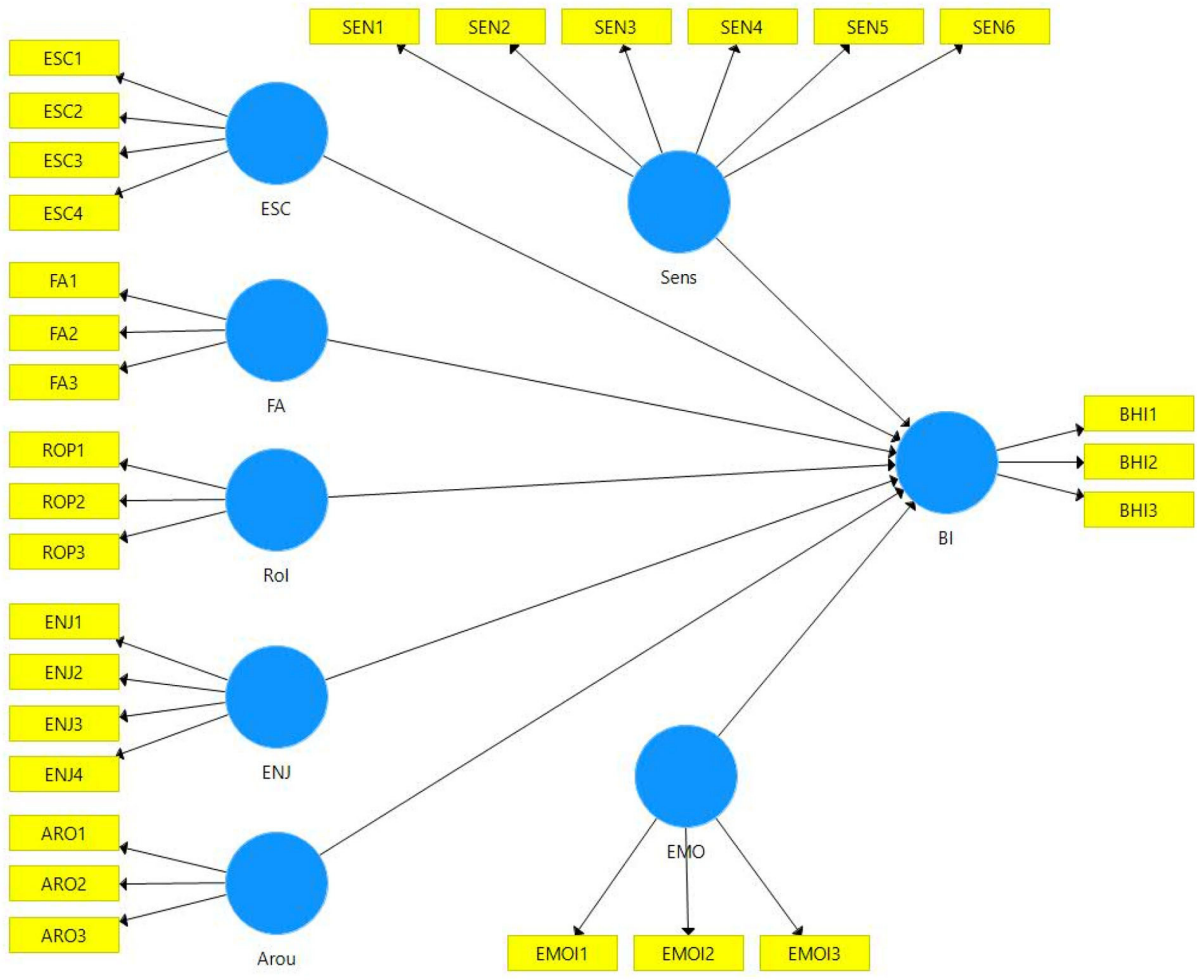
The study conceptual model.

This study utilized the partial least squares-structural equation modeling (PLS-SEM) for testing the proposed relationships, as shown in Figure 1. We applied the PLS-SEM technique due to various reasons. For instance, it supports the study's exploratory nature in which the researchers are more interested in testing the preliminary hypothesis (Hair et al., 2019; Abbasi et al., 2020b). Second, PLS-SEM is more suitable for theory development as we aim to investigate how playful-consumption experiences influence PUBG users' behavioral intention to play the game (Hair et al., 2017). Third, PLS-SEM is appropriate for prediction-oriented studies and can better predict the overall model through several quality checks comprising R^2^ (coefficient of determination), Q^2^ (predictive relevance), and PLSpredict, referring to out-of-sample prediction (Shmueli et al., 2019).

## Data analyses

Employing the PLS-SEM approach, we performed the PLS-SEM analyses in two main stages. In the first stage, we assessed the measurement model based on the model specification shown in Figure 1, which represents that all the constructs are reflectively measured. This is because the direction of causality is from construct to measure; hence, it is expected to correlate with indicators and internal consistency (Jarvis et al., 2003; MacKenzie et al., 2005).

Hair et al. (2019) recommended assessing the reflective constructs via indicator reliability, composite reliability (C.R.), and convergent validity (Hair et al., 2017, 2019). For indicator reliability, outer loading should be 0.70 or must exceed the value of 0.40. Cronbach's Alpha, C.R., and Dillon-Goldstein's Rho should surpass the threshold value of 0.70 to demonstrate construct reliability. For convergent validity, the average variance extracted (AVE) must be at least 0.5. We evaluated the reflective constructs and found that item loading exceeded the value of 0.70, reliabilities surpassed the value of 0.70, and AVE values met the minimum threshold value of 0.50. Hence, all constructs have met the criteria of being reliable and sound, see Table 2.

**Table 2:** Construct reliability and validity.

**Study variables**	**Items**	**Items-loadings**	**Cronbach's alpha**	**rho_A**	**Composite reliability**	**Average variance extracted (AVE)**
Escapism	ESC-1	0.821	0.857	0.866	0.904	0.702
	ESC-2	0.894				
	ESC-3	0.883				
	ESC-4	0.744				
Fantasy	FA-1	0.788	0.818	0.839	0.892	0.735
	FA-2	0.914				
	FA-3	0.865				
Role-projection	ROP-1	0.923	0.898	0.901	0.937	0.831
	ROP-2	0.901				
	ROP-3	0.91				
Enjoyment	ENJ-1	0.662	0.883	0.904	0.922	0.751
	ENJ-2	0.914				
	ENJ-3	0.937				
	ENJ-4	0.923				
Arousal	ARO-1	0.896	0.859	0.865	0.914	0.78
	ARO-2	0.886				
	ARO-3	0.868				
Emotional	EMOI-1	0.877	0.843	0.844	0.906	0.762
	EMOI-2	0.903				
	EMOI-3	0.838				
Sensory experience	SEN-1	0.706	0.846	0.858	0.885	0.563
	SEN-2	0.741				
	SEN-3	0.734				
	SEN-4	0.739				
	SEN-5	0.77				
	SEN-6	0.808				
Behavioral intention	BHI-1	0.945	0.839	0.891	0.905	0.763
	BHI-2	0.945				
	BHI-3	0.708				

Finally, the last aspect of the reflective measurement model assessment that merits research attention is discriminant validity. Discriminant validity is evaluated using the heterotrait-monotrait (HTMT) ratio correlation. Henseler et al. (2015) argue that HTMT ratios are acceptable below 0.90 and ideal below 0.85. Table 3 shows the corresponding results. Based on these research findings, we can confidently conclude that all constructs have achieved satisfactory values.

**Table 3:** Discriminant validity.

	**Arou**	**BI**	**EMO**	**ENJ**	**ESC**	**FA**	**Rol**	**Sens**
**Arou**								
BI	0.727							
EMO	0.742	0.731						
ENJ	0.586	0.716	0.709					
ESC	0.577	0.672	0.535	0.576				
FA	0.288	0.159	0.257	0.1	0.405			
Rol	0.483	0.554	0.429	0.52	0.736	0.417		
Sens	0.644	0.633	0.619	0.556	0.581	0.339	0.494	

Arou, Arousal; BI, Behavioral Intention; EMO, Emotional Involvement; ENJ, Enjoyment; ESC, Escapism; FA, Fantasy; Rol, Role projection; Sens, Sensory Experience.

If HTMT 0.90 = Good, HTMT 0.85 = Best.

Once we achieved the reflective measurement model's satisfactory results, we proceeded to assess the structural model for hypotheses testing. To evaluate the structural model, we followed Hair et al. (2020). We used the following quality checks (e.g., estimating the size and path-coefficients): R^2^ of the endogenous variable (in-sample prediction), f^2^ effect size (in-sample prediction), predictive relevance Q^2^ (primarily in-sample prediction), and PLSpredict (out-of-sample prediction). This study analyzed the structural model by performing the bootstrap samples of 5,000 and presented the results in Table 4.

**Table 4:** Structural model analysis.

	**Original sample (O)**	**Standard deviation (STDEV)**	**T Statistics (|O/STDEV|)**	***P*-Values**	**f2**	**R2**		**Decision**
H1: ESC -> BI	0.176	0.065	2.714	0.003	0.037			Supported
H2: FA -> BI	−0.113	0.048	2.362	0.009	0.026			Rejected
H3: Rol -> BI	0.092	0.064	1.439	0.075	0.011			Rejected
H4: ENJ -> BI	0.194	0.071	2.726	0.003	0.047			Supported
H5: EMO -> BI	0.202	0.076	2.638	0.004	0.047			Supported
H6: Arou -> BI	0.225	0.072	3.115	0.001	0.063			Supported
H7: Sens -> BI	0.137	0.06	2.292	0.011	0.026	0.596	0.437	Supported

H2: rejected due to negative path-coefficient and H3: rejected due to not meeting the minimum threshold value of effect size i.e., 0.02, which refers to a small effect.

**Figure 2:**
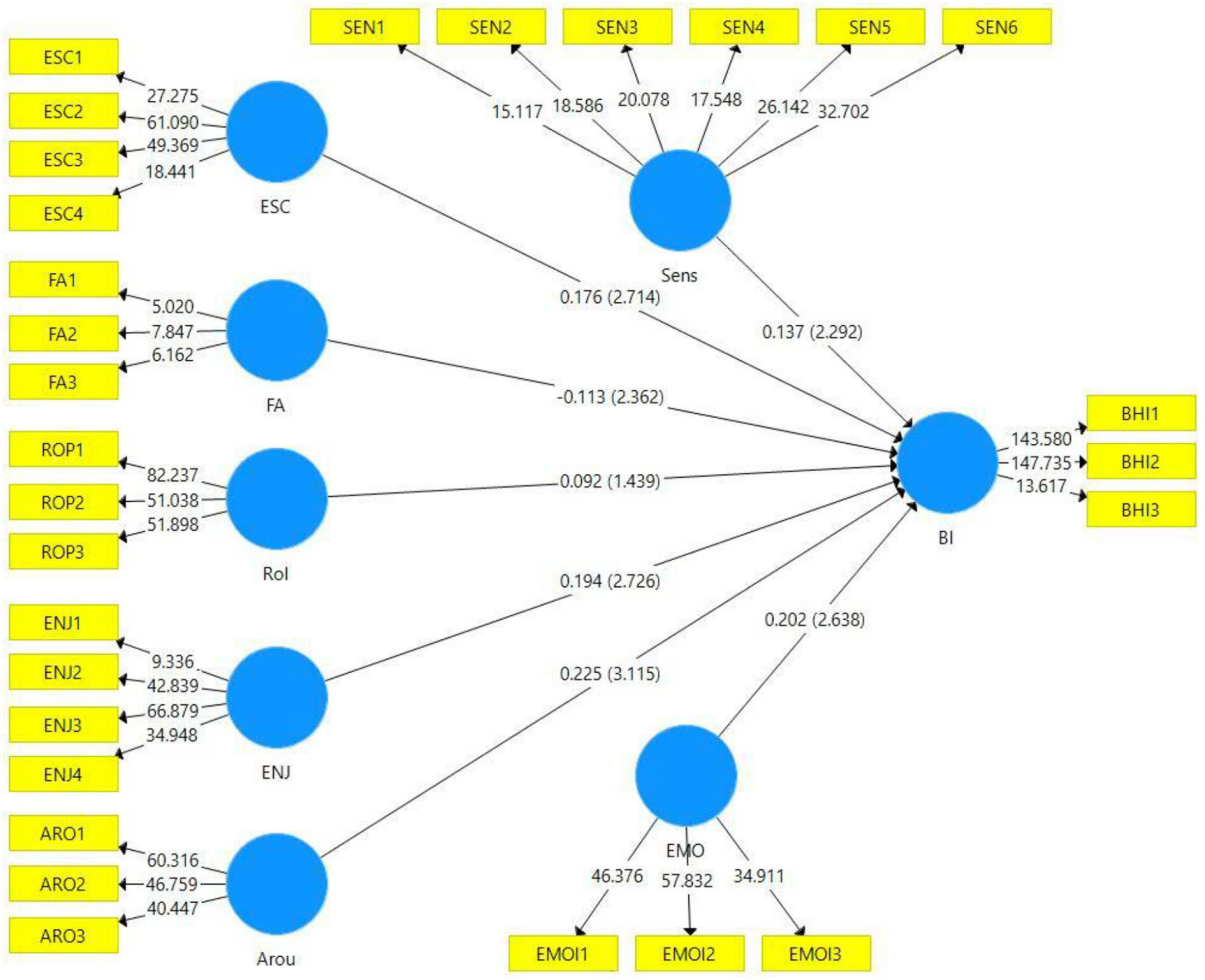
The structural model assessment.

Refer to Table 4 and Figure 2 for study results. The results in Table 4 showed a positive relationship between escapism and behavioral intention to play PUBG game with a path coefficient of 0.176, *P*-value of 0.003, t-value of 2.714, and f^2^ = 0.037. These results support our first hypothesis, H1. A negative relationship has been found between fantasy and behavioral intention to play PUBG game with a path coefficient of −0.113, *P*-value of 0.009, t-value of 2.362, and f^2^ = 0.026. Hence, we reject the H2 due to the negative path coefficient. Role-projection has failed to impact the behavioral intention with the value of path coefficient of 0.092, *P*-value of 0.075, t-value of 1.439, and f^2^ = 0.011. Enjoyment, emotional involvement, arousal, and sensory experience prove to significantly determine gamers' behavioral intention to play the PUBG game; see Table 4 for detailed values meeting the recommended threshold values.

We also calculated R2 and Q2 for the endogenous construct, which results in 0.596 and 0.437. The value of R2 exceeds the value of 0.26 (i.e., large) and represents that model is satisfactory (Cohen, 1988). The Q2 value also surpasses the zero value, which is a good sign of having high predictive relevance of the model (Hair et al., 2020).

The final step of the structural model assessment is out-of-sample prediction. To measure the out-of-sample prediction, we applied the PLSpredict as suggested by Shmueli et al. (2019) to strengthen the result produced by PLS-SEM. Shmueli et al. (2019) recommend that the researcher perform the PLSpredict on the outcome variable (i.e., the behavioral intention in our study). We analyzed the PLSpredict using the subdivision of the sample into 10 with 10 reiterations. The results are produced in Table 5. At times of interpreting the results of PLSpredict, we have to compare the PLS results with LM (linear regression model). If the values of LM mean absolute error (MAE) is higher for the outcome construct items than PLS-MAE, the model has high prediction power (Shmueli et al., 2019). The study results show that LM-MAE is higher than PLS-MAE, which explicates that the model has high predictive power.

**Table 5:** PLSPredict results.

**Dependent Variable**	**PLS-SEM Analysis**	**L.M**.							**Comparison**
**Items**	**RMSE**	**MAE**	**MAPE**	**Q^2^_predict**	**RMSE**	**MAE**	**MAPE**	**Q^2^_predict**	**PLS-LM (RMSE)**
BHI1	0.698	0.527	17.952	0.521	0.736	0.547	18.655	0.468	−0.038
BHI2	0.679	0.507	17.313	0.533	0.737	0.546	18.666	0.45	−0.058
BHI3	0.921	0.736	25.433	0.217	0.966	0.767	26.222	0.138	−0.045

## Discussion

This study explores different playful-consumption experience factors influencing behavioral intentions playing PUBG, a multiplayer online battle arena game.

Escapism was found to have a positive relationship with behavioral intention. This finding is consistent with previous research where escapism positively impacted behavioral intention to play games belonging to the same genre as PUBG (Wu and Holsapple, 2014; Abbasi et al., 2019a). This relationship may be explained using the rationale that players seek an escape from their real-life surroundings by indulging in videogames. PUBG provides users with an alternate environment that helps them elude real-life worries and tensions. Fantasy was observed to have a negative impact on behavioral intention. This is inconsistent with the idea put forth by Hirschman (1983) that users who indulge deeply in fantasies are more likely to consume products that are conducive to fantasies or the act of fantasizing. Thereby, this result may imply that PUBG creates a multi-sensory environment that is either not in line with the fantasies PUBG players have or does not support an individual's ability to fantasize (Wu and Holsapple, 2014). Emotional involvement, enjoyment, and arousal were also found to impact behavioral intention positively. These findings are consistent with previous research (Wu and Holsapple, 2014; Hsiao et al., 2018). This may be because arousal and enjoyment add to emotional engagement, which results in positive emotions when playing PUBG. As a result of this emotional response to playing PUBG, their intention to play may be bolstered.

However, the study found insignificant results for H3, reflecting that role projection factors fail to have a positive association with behavioral intention. This is also consistent with the findings of Wu and Holsapple (2014). The rejection of these hypotheses could be explained by the fact that PUBG allows users to customize their avatars to only a certain extent (Klang, 2004). As a result, PUBG players may not relate to the roles (pertaining to the avatar and narratives) they have been assigned and observed in the game. Furthermore, unlike other media forms, PUBG does not present characters in a three-dimensional manner, explaining the positive but insignificant relationship.

The sensory experience was also found to impact behavioral intention positively. Sensory experience regarding smartphone usage was also found to positively correlate with children's subjective well-being (Abbasi et al., 2020c) and consumer videogame engagement (Abbasi et al., 2019a). Sensory experience is an important variable that is not often studied in the context of pleasure-oriented I.S. since prior studies have ignored this relationship (Li et al., 2015; Patzer et al., 2020). Sensory experiences can lead to upbeat feelings, which may strengthen the desire to play PUBG, thereby resulting in a significant relationship (Almeida et al., 2016).

### Theoretical implications

In theoretical terms, this study applies the marketing theory of hedonic consumption and UGT to understand the antecedents that lead to the formulation of BR games' behavioral intentions, in particular of PUBG. Intent generally transforms into more prevalent usage behaviors because it satisfies the hedonistic goals of attaining pleasure. Most of the existing research had only focused upon only a few hedonic factors, such as enjoyment, fantasy, and escapism, which has not been enough in explaining players' behavioral intentions when playing BR games. This paper, however, gauges numerous playful-consumption experience factors, such as escapism, emotional involvement, sensory experience, enjoyment, arousal, role-projection, and fantasy, to determine players' behavioral intention to PUBG. By focusing the research on PUBG, a relevant contemporary BR game, this research can advance the theoretical development of hedonic consumption and UGT through empirical insights derived from actual players. Furthermore, while existing literature predicted children's subjective well-being in terms of their smartphone usage (Abbasi et al., 2020c), videogame consumer engagement (Abbasi et al., 2019a), and attitude formation toward playing the PUBG game (Hollebeek et al., 2022), by using the hedonic theory, this research extends its use to determining behavioral intention to play PUBG. Similarly, it extends and develops the understanding of the UGT to the area of behavioral intentions as well. This research also creates a novel conceptual framework where it combines features of video games, virtual reality, and social media to determine influence on behavioral intentions of pleasure-oriented I.S.

### Practical implications

Since video games have emerged as a popular industry within pleasure-oriented I.S., there is a need to expand our understanding of users' behavioral intentions to play video games. By evaluating factors that influence behavioral intentions to play video games, upcoming games can be tailored with design features that can lead to increased usage (Korhonen et al., 2009). Such research findings could also be aimed at addressing additional issues such as video game addiction. This research would carry direct implications for the user experience researchers, game development companies, and video game consumers because it would help them understand the factors that influence user engagement with video games.

For game developers and firms, this research's findings can highlight the significance of experiential dimensions that augment users' behavioral intention to play PUBG. They can use the findings about players' playful consumption experiences to develop games with customized game environments to increase game engagement and mass persuasion. They can prioritize the relevance of certain consumption experience factors, such as escapism, emotional involvement, sensory experience, enjoyment, and arousal, over other factors, such as role-projection and fantasy. Developers can prioritize a combination of these factors to cater to different audiences. For example, they can reduce elements of fantasy and increase elements of emotional involvement to appeal to an audience that prefers PUBG over less realistic alternatives. In keeping with our findings, these elements are not significantly related to the continued intention to play the game. This could help practitioners emphasize on aspects that are valued by players during their gameplay.

Game developers and other practitioners could use this research to further enhance the user experience by ensuring that the game satisfies the goals set by consumers. If users are looking to immerse themselves in a game and escape their reality, then pleasure-oriented I.S. such as communication with other players could be made more sensational and emotional to allow better expression. It may also help developers understand how escapism does not need to come from fantasy for they can use realistic elements to create alternate realities that detach an individual from their everyday experiences. Furthermore, this research can aid in helping developers understand the importance of creating a sensory experience for the users by including esthetic elements which can captivate the players and help them immerse themselves fully in the game for an enjoyable emotional experience, detached from reality. By tapping into numerous factors that influence behavioral intention when playing BR games, such as PUBG, including sensory experiences, emotional involvement, and escapism, this research opens the door for further research into different sensory and emotional elements that can be incorporated by game developers into their work.

This research can help researchers in better implementing the Technology Acceptance Model (TAM) across multiple systems by contributing to their understanding of the difference between productivity-oriented and pleasure-oriented I.S. Productivity-oriented I.S is able to enhance the user experience by focusing upon the intention and extrinsic motivations of the user behind their practical use of the system. Thus, the utilitarian perspective might be more useful. While the pleasure-oriented I.S. is based more on intrinsic motivations, hedonic theory and UGT might be more appropriate.

However, this research study has some clear limitations. First, the results of the study are not generalizable beyond the ages of 16 and 40 years. We chose this sample because it was representative of a population that mostly plays the PUBG game. It was also hard for us as researchers to find PUBG gamers that did not fit this age bracket. Furthermore, the participants were only male PUBG gamers; an equal gender population would have been desirable as that would have strengthened the results and extended the generalizability to the female participants. However, again it was hard for the research to find a reasonable sample of female PUBG players. Finally, the exploration model and hypotheses are tested in a specific setting, therefore additional studies are required on different demographics to certify the generalizability of the results beyond the specific context tested in the experiment. The playful-consumption experience model was applied in the PUBG game and it can be expanded to different mediums such as virtual reality games or any digital technologies comprising virtual, augmented, and extended realities. We only focused on the behavioral intention perspective using the U&G theory. Future studies can employ the technology acceptance model to study the role of playful-consumption experiences in predicting performance expectancy, ease of use, perceived usefulness, effort expectancy, and other factors.

To summarize, our research findings suggest that some sensory, imaginal, and emotional experiences have a strong effect on users' behavioral intentions to play the PUBG game. Factors such as escapism, emotional involvement, enjoyment, and arousal significantly affect the behavioral intentions of playing PUBG. Our findings further suggest that role-projection and fantasy negatively impacted consumers' intention to play PUBG game. These findings offer significant implications for modern game development companies and I.S researchers.

## Data availability statement

The datasets for this study are available on request to the corresponding author.

## Ethics statement

The study was reviewed and approved by IRC for Finance and Digital Economy, KFUPM Business School, King Fahd University of Petroleum and Minerals. The participants provided their written informed consent prior to participating in this study.

## Author contributions

UR and AA worked on the idea development and conceptual design. MS and RI worked on the literature review and improved the contents of the paper. AA worked on the data collection and analyses. HH edited the complete manuscript, extended implications, and edited the literature review. UR worked on the multiple revisions rounds and addressed all reviewer concerns. All authors contributed to this paper and approved the submitted draft.

## Conflict of interest

The authors declare that the research was conducted in the absence of any commercial or financial relationships that could be construed as a potential conflict of interest.

## Publisher's note

All claims expressed in this article are solely those of the authors and do not necessarily represent those of their affiliated organizations, or those of the publisher, the editors and the reviewers. Any product that may be evaluated in this article, or claim that may be made by its manufacturer, is not guaranteed or endorsed by the publisher.

## Figures and Tables

**TABLE A1:** 

**Construct**	**Items**	**Statement**
Escapism	Es1	Playing the PUBG game gets me away from the reality.
	Es2	Playing the PUBG game gets me away from the problems and pressures.
	Es3	Playing the PUBG game helps me escape from things that are unpleasant and worrying.
	Es4	Playing the PUBG game makes me feel like I am in a different world of reality.
Fantasy	Fa1	Playing the PUBG game helps me construct fantasies.
	Fa2r	Playing the PUBG game does not stimulate my imagination.
	Fa3	Playing the PUBG game helps me create daydreams.
	Fa4	Playing the PUBG game helps me augment reality.
Role-projection	Rp1	Playing the PUBG game enables me to project myself into a particular role.
	Rp2	Playing the PUBG game enables me to project myself into a particular character.
	Rp3	Playing the PUBG game enables me to project myself into a particular task.
	Rp4	Playing the PUBG game enables me to project myself into someone else.
Enjoyment	En1r	Playing the PUBG game is not really fun.
	En2	Playing the PUBG game provides me with a lot of enjoyment.
	En3	Playing the PUBG game is enjoyable.
	En4	I enjoy playing the PUBG game.
Emotional involvement	EI1	When I am playing the PUBG game, I feel carried off.
	EI2	When I am playing the PUBG game, I feel as I am part of this video-game.
	EI3	When I am playing the PUBG game, I feel deeply about this video-game.
	EI4	When I am playing the PUBG game, I get into this video-game.
	EI5	After I finish playing the PUBG game, I may carry the video-game playing experience with me for a while.
Arousal	Ar1	Playing the PUBG game makes me stimulated.
	Ar2r	Playing the PUBG game does not make me excited.
	Ar3	Playing the PUBG game makes me inspired.
	Ar4	Playing the PUBG game makes me wide-awake.
	Ar5	Playing the PUBG game makes me motivated.
Sensory experience	SE1	Playing the PUBG game influences my physical movement.
	SE2	My body adapts sudden actions, as a reaction to certain situations in the video-game *(e.g., tries to move my hand in the direction of gun I am pointing in the game etc*.).
	SE3	The peripheral video-gaming device (joy-stick, joy-pad, and other accessories) makes me actually feel the physical experience of the game.
	SE4	The video-game music stimulates my emotions to adapt and react accordingly *(e.g., I play aggressively with aggressive music, I play calmly with soft music and react in fear to a horror music etc.)*.
	SE5	The scenic beauty of the video-game is aesthetically appealing me.
	SE6	The visuals of the video-game fill my appetite for unique and different structure, shapes and design.
Behavioral intention	BI-1	I intend to play the PUBG game in the future
	BI-2	I intend to play the PUBG game as regularly as I do now
	BI-3	I intend to play the PUBG game as much as possible in the future
